# Transition to absence seizures and the role of GABA_A_ receptors

**DOI:** 10.1016/j.eplepsyres.2011.07.011

**Published:** 2011-12

**Authors:** Vincenzo Crunelli, David W. Cope, John R. Terry

**Affiliations:** a Neuroscience Division, School of Biosciences, Cardiff University, Museum Avenue, Cardiff CF10 3US, UK; b Department of Automatic Control and Systems Engineering, University of Sheffield, S10 2TN, UK; c Sheffield Institute for Translational Neuroscience, 452a Glossop Road, Sheffield S10 2HQ, UK

**Keywords:** Phasic GABA_A_ inhibition, Tonic GABA_A_ inhibition, GABA_B_ receptors, GHB, GAERS, Mathematical modelling

## Abstract

Absence seizures appear to be initiated in a putative cortical ‘initiation site’ by the expression of medium-amplitude 5–9 Hz oscillations, which may in part be due to a decreased phasic GABA_A_ receptor function. These oscillations rapidly spread to other cortical areas and to the thalamus, leading to fully developed generalized spike and wave discharges. In thalamocortical neurons of genetic models, phasic GABA_A_ inhibition is either unchanged or increased, whereas tonic GABA_A_ inhibition is increased both in genetic and pharmacological models. This enhanced tonic inhibition is required for absence seizure generation, and in genetic models it results from a malfunction in the astrocytic GABA transporter GAT-1. Contradictory results from inbred and transgenic animals still do not allow us to draw firm conclusions on changes in phasic GABA_A_ inhibition in the GABAergic neurons of the nucleus reticularis thalami. Mathematical modelling may enhance our understanding of these competing hypotheses, by permitting investigations of their mechanistic aspects, hence enabling a greater understanding of the processes underlying seizure generation and evolution.

## Introduction

A typical absence is a non-convulsive epileptic seizure that is characterized by impairment of consciousness which occurs concomitantly with a generalized, bilaterally synchronous ‘spike (or polyspike) and slow wave discharge’ (SWD) at 2.5–4 Hz in the EEG (Crunelli and Leresche, 2002; Blumenfeld, 2005). Absence seizures are part of the multi-faceted clinical and EEG presentation of many idiopathic generalized epilepsies, though in childhood absence epilepsy these seizures are the only neurological symptom and are not accompanied by either metabolic, neuropathological or other neurological deficits (Crunelli and Leresche, 2002; Blumenfeld, 2005; Snead, 1995). Absence seizures are genetically determined and originate from abnormal electrical activity in reciprocally connected thalamic and cortical territories, i.e. in what is generally referred to as thalamo-cortical networks, with little or no involvement of other brain areas (Holmes et al., 2004; Hamandi et al., 2006; Westmijse et al., 2009; Bai et al., 2010; Szaflarski et al., 2010). Key cellular elements of thalamo-cortical networks include pyramidal cells and interneurons of different cortical layers, the thalamocortical (TC) neurons of sensory thalamic nuclei and their main inhibitory input, i.e. the GABAergic neurons of the nucleus reticularis thalami (NRT).

## The putative cortical ‘initiation site’ and the transition to SWDs

The notion that a typical absence seizure is ‘generalized’ from the very start of the SWD has been recently challenged by high-density EEG and imaging studies showing that the onset of an absence seizure in humans is associated with paroxysmal activation of discrete, most often frontal and parietal cortical regions, spreading then to other cortical regions and to the thalamus (Holmes et al., 2004; Hamandi et al., 2006; Westmijse et al., 2009; Bai et al., 2010). The presence of a putative cortical ‘initition site’ had previously been suggested on the basis of results in genetic rat models of absence seizures, where differently from human absences, however, it appears to be localized in the perioral region of the primary somatosensory cortex (Meeren et al., 2002; Polack et al., 2007). Indeed, direct application of the anti-absence drug ethosuximide in this cortical region, but not 1 mm away from it (i.e. in the primary motor cortex), readily abolishes absence seizures in freely moving Genetic Absence Epilepsy Rats from Strasbourg (GAERS) rats, a well-established inbred model of this type of epilepsy (Manning et al., 2004). Another significant observation of one of these studies was that the manifestation of the behavioural component of an absence seizure requires electrographic abnormalities to be present both in cortex and thalamus (Polack et al., 2007).

Differently from convulsive epilepsy, the transition from normal electrographic activity to SWDs has not received a lot of attention in either clinical or experimental studies. One of the notable exceptions to this has been the analysis of intracellularly recorded TC and NRT neurons in GAERS and its comparison with their non-epileptic control (NEC) rat strain (Pinault et al., 2001). In this study, it was shown that the majority of SWDs appear to develop from medium-amplitude oscillations at 5–9 Hz, which relatively rapidly (within 1–2 s) evolve into the larger amplitude oscillations with a clearly defined waveform of spike and wave components that are characteristic of the SWDs of absence seizures. Importantly, these medium-amplitude 5–9 Hz oscillations are distinct from sleep-spindles and also occur in the NEC strain, indicating that they are not, by themselves, sufficient to initiate SWDs but require the presence of abnormal intrinsic and/or synaptic mechanisms to develop into absence paroxysms.

## Mechanistic models of seizure initiation and evolution

**Figure 1:**
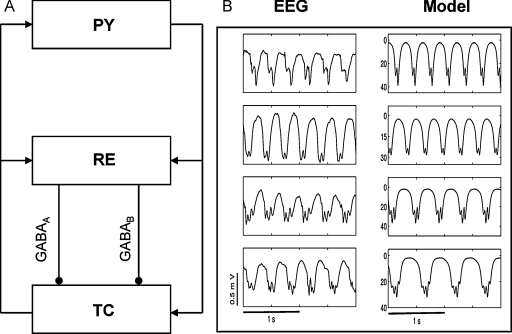
Macroscopic modelling of EEG activity. (A) Schematic of the neural mass model considered in Marten et al. (2009a). The model considers interactions between populations of excitatory pyramidal (PY) neurons in the cortex and inhibitory reticular (RE) neurons and excitatory thalamocortical (TC) neurons in the thalamus. Inhibition between RE and TC populations is mediated by both GABA_A_ and GABA_B_ receptors. (B) Comparison of the model output and exemplar EEG dynamics from patients with SWDs within our clinical database. Within the model, adjusting the ratio of inhibition mediated by GABA_A_ and GABA_B_ causes the appearance of additional spikes within a spike–wave cycle, which is a characteristic feature of seizure evolution of subjects with absence epilepsy. Theoretically, this transition is due to effectively adjusting the timescale of inhibition mediated by the two receptor types.

To elucidate the mechanisms responsible for seizure initiation and evolution there has been much recent interest in the use of mathematical models, the output of which can be directly related to experimental or clinical observables. Inspired by the pioneering research of Freeman (1975) and Lopes da Silva (Lopes da Silva et al., 1974), several researchers have developed models suggesting potential mechanisms underpinning the generation of rhythms of activity observed in the EEG during both normal and seizure states (see Deco et al. (2008) for a comprehensive review). Given the importance of both the cortico-thalamic loop (Destexhe and Sejnowski, 2001) and GABA receptors in the generation of abnormal waveforms observed during seizures these physiological mechansisms were incorporated into a model of the dynamics of large-scale brain activity (Robinson et al., 2002). It was shown that – at a macroscopic level – an interplay between the strength of cortico-thalamic connections and GABA receptor mediated inhibition led to a cascade of transitions corresponding to seizure initiation and evolution (Rodrigues et al., 2006). More recent work has shown that the appearance of additional dynamic features (such as poly-spike complexes) can occur due to a complex relationship between the levels GABA_A_ and GABA_B_ receptor mediated inhibition of thalamo-cortical cells (Marten et al., 2009a, 2009b). A schematic diagram of the considered neural mass model and its output is presented in Fig. 1, where a clear resemblance to clinically recorded EEG activity can be observed. Linking these macroscopic models to experimental observations by incorporating further physiological details (such as those described within the present article) may enable an enhanced elucidation of the mechanisms responsible for characteristic features of experimental recordings during SWDs (for example, poly-spike and wave complexes or the frequency slowing during seizure evolution).

## Phasic GABA_A_ receptor-mediated inhibition

Abnormalities in synaptic GABA_A_ receptors (GABA_A_Rs) have undoubtedly been of primary significance among the various human molecular genetic alterations (MacDonald et al., 2010) that have in recent years provided support for the idea that absence seizures are channelopathies (Noebels, 2003). Functional analysis of these mutant proteins expressed in heterologous systems has shown that they all bring about a decrease in GABA response (MacDonald et al., 2010). In particular, mice where one of these human mutations, i.e. the γ2(R43Q), has been expressed by homologous recombination show spontaneous absence seizures and exhibit a reduction in miniature IPSCs (mIPSCs) in cortical layer 2/3 pyramidal cells but not in TC or NRT neurons compared to age-matched wildtype littermates (Tan et al., 2007). This clearly indicates that the downstream abnormalities of a human genetic variant associated with absence seizures can be brain region-specific. However, no changes in GABA_A_ IPSCs properties are detected in layer 2/3 pyramidal and non-pyramidal neurons of GAERS compared to non-epileptic rats (Bessaïh et al., 2006). These contradictory data between rats and mice models of absence seizures may reflect the fact that the latter models often show other neurological deficits.

As far as the thalamus is concerned, both in felines that show spontaneous or cortical bicuculline-induced SWDs (Steriade and Contreras, 1995) as well as in the well-established GAERS model (Pinault et al., 1998) the vast majority (60 and 94%, respectively) of TC neurons recorded *in vivo* during SWDs exhibit bursts of GABA_A_ IPSPs, each tightly synchronized with the EEG spike and wave complex. Indeed, the rise time, amplitude, frequency and decay time constant of mIPSCs and spontaneous IPSCs (sIPSCs) measured in TC neurons *in vitro* are not different between GAERS and NEC, and paired pulse depression of evoked IPSCs is also similar between the two strains (Bessaïh et al., 2006). As mentioned earlier, no change in TC neuron mIPSCs are present in mice carrying the human γ2(R43Q) mutation, and no change in IPSC properties has been detected in TC neurons of β3 KO mice (Huntsman et al., 1999) which show absence seizures as part of a much more complex neurological phenotype, as well as in sIPSCs in lethargic, stargazer and tottering mice (Cope et al., 2009). In a similar manner to the genetic models, in the best-established pharmacological model of absence seizure, i.e. the γ-hydroxybutyric acid (GHB) model, a net increase in phasic GABA_A_ inhibition is observed in TC neurons of the ventrobasal thalamus (Gervasi et al., 2003). This is because at doses that elicits absence seizures GHB reversibly and dose-dependently decreases the amplitude of all sensory and corticothalamic EPSCs but only some IPSCs, thus leading to a net increase in phasic GABA_A_ inhibition. In summary, it is surprising that against this wealth of data both from genetic and pharmacological models, as well as from mice expressing the human γ2(R43Q) mutation, the current view (McCormick and Contreras, 2001; Destexhe and Sejnowski, 2003; Budde et al., 2006; Huguenard and McCormick, 2007; Beenhakker and Huguenard, 2009) of the pathophysiological mechanisms of TC neuron activity during absence seizures is still the one that is observed in brain slices in the presence of a GABA_A_R antagonist (von Krosigk et al., 1993; Bal et al., 1995; Kleiman-Weiner et al., 2009).

As far as NRT neurons are concerned, mIPSCs in GAERS have a higher frequency, a larger amplitude and a faster decay than those in age-matched NEC, and paired-pulse depression of evoked GABA_A_ IPSCs is significantly smaller in the former than the latter strain (Bessaïh et al., 2006). Moreover, an increase in mIPSC frequency is observed in NRT neurons of the absence seizure-prone DAB/2J mouse strain (Tan et al., 2008), whereas an almost complete disappearance of the α3 subunit has been reported in these GABAergic neurons from Wistar Albino Glaxo (WAG) rats, another well-established inbred model of absence epilepsy (Liu et al., 2007). However, α3 KO mice do not show spontaneous absence seizures and exibit a small reduction in GHB-elicited seizures, a result that has been interpreted as resulting from a powerful compensatory gain in phasic GABA_A_ inhibition in NRT neurons (Schofield et al., 2009). Further evidence for a pro-absence role of a decreased phasic GABA_A_R function in NRT neurons has also come from the observation of an increase in high frequency discharges at 3 Hz in β3 subunit KO mice, which show a massive reduction in sIPSP frequency and amplitude in NRT, but no change in TC neurons (Huntsman et al., 1999). Since these β3 KO mice exhibit a large variety of neurological deficits and are considered a model of Angelman's syndrome, it is difficult to unequivocally assign a causative role for this decreased intra-NRT inhibition in typical absence seizures. In summary, whereas in inbred models and models with spontaneous mutations there is either an increase or no change in intra-NRT phasic GABA_A_ inhibition, data from two transgenic mice suggest that a decrease in this NRT synaptic function has a pro-absence effect. Thus, one could speculate that abnormalities in intra-NRT phasic GABA_A_ inhibition are not a necessary condition for the expression of typical absence seizures, a view supported by the lack of changes in NRT IPSCs of mice carrying the human γ2(R43Q) mutation (Tan et al., 2007).

## Tonic GABA_A_R-mediated inhibition

**Figure 2:**
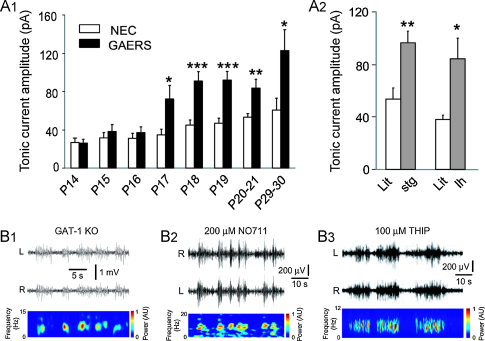
Enhanced tonic GABA_A_ current in TC neurons of absence epilepsy models and expression of SWDs following genetic or pharmacological block of the GABA transporter GAT-1. (A1) The amplitude of the tonic GABA_A_ current measured in TC neurons of the VB is about two-fold larger in GAERS compared to NEC at postnatal day (P) 17 and remains elevated well after the time of absence seizure onset in this model (i.e. around P20). (A2) Tonic GABA_A_ current in stargazer (stg) (P19–21) and lethargic (lh) (P27–30) mice and respective age-matched littermates (Lit). *p < 0.05, **p < 0.01 and ***p < 0.001, mutant *vs.* non-mutant animals. (B) Bilateral (L = left, R = right hemispheres) EEG traces showing the presence of spontaneous SWDs in a freely moving GAT-1 knockout (KO) mouse (B1), in a freely moving normal Wistar rat following direct thalamic injection of the selective GAT-1 blocker NO711 in the VB (B2), and in another freely moving normal Wistar rat following direct thalamic injection of the GABA_A_ δ-subunit selective agonist THIP in the VB (B3). Spectrograms of the R trace are illustrated below each EEG trace. Illustrated concentration of NO711 and THIP is that of the dialysis probe inlet.

It is now well established that GABA_A_R-mediated inhibition consists not only of a phasic component (i.e. the ‘classical’ IPSPs), that is generated by GABA interacting with synaptic GABA_A_Rs, but also of a tonic component (i.e. a persistent membrane hyperpolarization with increased conductance) that is due to GABA activation of perisynaptic or extrasynaptic GABA_A_Rs (Farrant and Nusser, 2005). Recent studies have shown that the tonic GABA_A_ current measured *in vitro* in TC neurons of the VB of different genetic models of absence seizures is enhanced compared to their respective control animals (Cope et al., 2009). This is true for a polygenic rat model (i.e. the GAERS) (Fig. 2A1) and for various mice models with known spontaneous monogenic mutations, including stargazer and lethargic mice (Fig. 2A2). In particular, there is a clear developmental profile of this increased GABAergic function since in GAERS up to postnatal day 16 the current is similar to that in the NEC strain but almost doubles in amplitude within the next 24 h (Fig. 2A1), and remains elevated well past the time of seizure onset (around postnatal day 20 in this strain). In contrast, no tonic GABA_A_ current is detected in the GABAergic NRT neurons of GAERS and their respective non-epileptic control strain (unpublished observation), as it is indeed the case in NRT neurons of normal Wistar rats (Cope et al., 2005) and mice (Belelli et al., 2005; Jia et al., 2005). The enhanced tonic GABA_A_ current of TC neurons of GAERS, and stargazer and lethargic mice is due to a malfunction of the GABA transporter GAT-1 (Cope et al., 2009), that in the thalamus has an astrocytic and not a neuronal location (Pow et al., 2005), and is supported by previous data indicating a reduced GABA uptake by GAT-1 (Sutch et al., 1999) and an increased level of extracellular GABA (Richards et al., 1995) in the VB of GAERS compared to NEC. Interestingly, GAT-1 activity is not compromised in GAERS dentate gyrus granule cells (Cope et al., 2009), an area that does not participate in the generation of absence seizures (Danober et al., 1998) and where the distribution of this transporter is primarily neuronal (De Biasi et al., 1998).

Systemic administration of THIP (4,5,6,7-tetrahydroisoxazolo[5,4-c]pyridin-3-ol), a selective agonist at δ subunit-containing extrasynaptic GABA_A_Rs (Brown et al., 2002), and systemic and intrathalamic application of GHB, a weak GABA_B_R agonist, elicit absence seizures in different species (Fariello and Golden, 1977; Snead, 1991). Application of either THIP or GHB *in vitro* leads to an increase in the tonic GABA_A_ current of TC neurons of normal Wistar rats (Cope et al., 2009). Whereas it is not surprising that THIP dose-dependently enhances the tonic GABA_A_ current in TC neurons of normal Wistar rats, one would not expect GHB, which does not bind to GABA_A_Rs and is believed to elicit absence seizures by activation of GABA_B_Rs (Crunelli et al., 2006), to have an effect on the tonic GABA_A_ current. However, this effect of GHB is indeed due to GABA_B_R activation since it is abolished by the selective GABA_B_R antagonist CGP55845 (Cope et al., 2009). Interestingly, application of CGP55845 alone decreases the tonic GABA_A_ current amplitude in TC neurons of GAERS, stargazer and lethargic mice (Cope et al., 2009), suggesting that facilitation of extrasynaptic GABA_A_R function by GABA_B_R activation contributes to the tonic current in these genetic models.

The enhanced tonic GABA_A_ current of TC neurons is not simply an epiphenomenon but plays a key role in the genesis of typical absence seizures. Thus, freely moving GAT-1 KO mice, which have an enhanced tonic GABA_A_ current in TC neurons, express ethosuximide-sensitive absence seizures (Fig. 2B1), and intrathalamic injection of no.711, a selective GAT-1 blocker, in normal Wistar rats initiates absence seizures (Fig. 2B2). Moreover, in GABA_A_R subunit KO mice, which exhibit a markedly reduced GABA_A_ inhibition in TC neurons (Cope et al., 2009; Herd et al., 2009), systemic administration of GHB fails to induce absence seizures (Cope et al., 2009). Similarly, the intrathalamic injection of a δ subunit-specific antisense oligodeoxynucleotide in GAERS decreases tonic GABA_A_ current and spontaneous absence seizures 1–2 days after injection (Cope et al., 2009). Finally, intrathalamic administration of the δ subunit-selective agonist THIP in normal Wistar rats elicits absence seizures in a concentration-dependent manner (Fig. 2B3) (Cope et al., 2009).

In summary, in both genetic and pharmacological models of absence seizures there is an increased tonic GABA_A_ inhibition in TC neurons, which in the genetic models is due to a GAT-1 malfunction in thalamic astrocytes. In addition, the enhanced tonic GABA_A_ inhibition in TC neurons is both necessary and sufficient for the generation of typical absence seizures. These data provide a mechanistic explanation for the aggravation of absence seizures that is observed in humans and experimental models following systemic and intrathalamic administration of drugs that increase GABA levels, including tiagabine and vigabatrine (Danober et al., 1998; Hosford and Wang, 1997; Perucca et al., 1998; Ettinger et al., 1999).

## Concluding remarks

The evidence reviewed here suggests that within a putative cortical ‘initiation site’ the expression of medium-amplitude 5–9 Hz oscillations, which may in part be due to a decreased phasic GABA_A_R function may lead to the expression of SWDs following entrainment of other cortical areas and the thalamus. The strong and highly synchronous cortical output powerfully excites the GABAergic neurons of the NRT (Slaght et al., 2002), leading in turn to bursts of IPSPs in TC neurons which override cortical excitation. Concomitantly, ambient GABA levels around TC neurons abnormally increase due to reduced GABA uptake by GAT-1, enhancing extrasynaptic GABA_A_R function. Enhanced tonic inhibition persistently hyperpolarizes the TC neurons and increases their membrane conductance, reducing the transfer of sensory inputs. Importantly, however, the rhythmic IPSP bursts entrain TC neuron output to each SW complex, thus maintaining paroxysmal activity in thalamo-cortical networks. To further elucidate the mechanisms underpinning the genesis and evolution of SWDs requires a closer integration between experimental and mechanistic modelling whereby predictive modelling informed by experimental data, can lead to new experimentally testable hypotheses.

## Meeting discussion

**Table:** 

*F. H. Lopes da Silva*: I want to make a small but important comment about the cortical ‘initiation site’ of absence seizures. The start of an absence seizure in cortex is only visible in the very first few hundred milliseconds of a seizure and is then immediately followed by a full entrainment of thalamus and cortex in the paroxysmal oscillations, so that it is no longer possible to identify which is the leading brain structure. Indeed, one of the reasons why this characteristic temporal development of SWDs had not been appreciated earlier was because appropriate analysis with high temporal resolution were not available. Also, the importance of this initiation site is supported by the finding of major abnormalities in Na+ channels and NMDA receptors.
*V. Crunelli*: I fully agree with your comment. It is indeed only at the very start of a SWD that one can detect such temporal separation between the two areas, but definitively not within each SW complex in the remaining part of the seizure.
*F. H. Lopes da Silva*: You only briefly commented on the role of GABA_B_ receptors in absence seizures, mainly in relation to the extrasynaptic GABA_A_ receptors. Can you tell us more?
*V. Crunelli*: Yes, I showed you that GHB increases tonic GABA_A_ current by an action on GABA_B_ receptors, and that selective GABA_B_ antagonists decrease this current in both GAERS and stargazer mice. We also have unpublished data showing that the selective GABA_B_ agonist baclofen does indeed increase the tonic current in TC neurons, suggesting the possibility of a direct cross-talk between GABA_B_ and GABA_A_ receptors. Our current view, therefore, is that it is possible that the pro- and anti-absence actions of GABA_B_ agonists and antagonists may be mediated both by changes in tonic GABA_A_ inhibition and by the other ‘classical’ GABA_B_R-mediated effectors, i.e. decrease in presynaptic release and generation of GABA_B_ IPSPs.
*F. H. Lopes da Silva*: I liked very much your model of absence seizure generation. One point I want to stress is that the possiblities you mentioned are not mutually exclusive. In the brain, of either experimental animals or patients, you have a set of abnormal parameters that it is sufficient for some random noise to change the dynamics of the system leading to the generation of seizures. This, however, does not exclude that you may also have changes in the modulation of these parameters such as for instance the cholinergic input which after all is very important in determining the behavioural state in which absence seizures may occur.
*J.R. Terry*: Indeed our modelling work has shown that two routes to seizure generation are permissable within the same model. As you highlight model parameters may be in a region of parameter space in which there exists ‘bistability’ whereby noise may switch you from an apparently healthy state to that of a seizure. Alternatively gradually changes in parameters can also correspond to a transition from apparently normal EEG dynamics to those associated with absence seizures. This latter possibility is particularly appealing since there is strong evidence from the recordings of human subjects that dynamic features of the waveform (such as the appearance of spikes) evolves over the course of a seizure and this is a consistent observation across multiple seizures from the same subject. Mechanistic modelling provides an ideal framework to explore competing hypotheses for the generation and evolution of seizures in this context.

## Figures and Tables

(B) Reproduced with permission from Marten et al. (2009a).

(A1, A2, and B1–B3) Reproduced with permission from Cope et al. (2009).
